# dialogi: Utilising NLP With Chemical and Disease Similarities to Drive the Identification of Drug-Induced Liver Injury Literature

**DOI:** 10.3389/fgene.2022.894209

**Published:** 2022-08-09

**Authors:** Nicholas M. Katritsis, Anika Liu, Gehad Youssef, Sanjay Rathee, Méabh MacMahon, Woochang Hwang, Lilly Wollman, Namshik Han

**Affiliations:** ^1^ Milner Therapeutics Institute, University of Cambridge, Cambridge, United Kingdom; ^2^ Department of Chemical Engineering and Biotechnology, University of Cambridge, Cambridge, United Kingdom; ^3^ Department of Chemistry, Centre for Molecular Informatics, University of Cambridge, Cambridge, United Kingdom; ^4^ Centre for Therapeutics Discovery, LifeArc, Stevenage, United Kingdom; ^5^ Cambridge Centre for AI in Medicine, University of Cambridge, Cambridge, United Kingdom

**Keywords:** drug-induced liver injury (DILI, ), natural language processing (NLP, ), neural network, disease similarity, drug similarity

## Abstract

Drug-Induced Liver Injury (DILI), despite its low occurrence rate, can cause severe side effects or even lead to death. Thus, it is one of the leading causes for terminating the development of new, and restricting the use of already-circulating, drugs. Moreover, its multifactorial nature, combined with a clinical presentation that often mimics other liver diseases, complicate the identification of DILI-related (or “positive”) literature, which remains the main medium for sourcing results from the clinical practice and experimental studies. This work–contributing to the “Literature AI for DILI Challenge” of the Critical Assessment of Massive Data Analysis (CAMDA) 2021– presents an automated pipeline for distinguishing between DILI-positive and negative publications. We used Natural Language Processing (NLP) to filter out the uninformative parts of a text, and identify and extract mentions of chemicals and diseases. We combined that information with small-molecule and disease embeddings, which are capable of capturing chemical and disease similarities, to improve classification performance. The former were directly sourced from the Chemical Checker (CC). For the latter, we collected data that encode different aspects of disease similarity from the National Library of Medicine’s (NLM) Medical Subject Headings (MeSH) thesaurus and the Comparative Toxicogenomics Database (CTD). Following a similar procedure as the one used in the CC, vector representations for diseases were learnt and evaluated. Two Neural Network (NN) classifiers were developed: a baseline model that accepts texts as input and an augmented, extended, model that also utilises chemical and disease embeddings. We trained, validated, and tested the classifiers through a Nested Cross-Validation (NCV) scheme with 10 outer and 5 inner folds. During this, the baseline and extended models performed virtually identically, with F_1_-scores of 95.04 ± 0.61% and 94.80 ± 0.41%, respectively. Upon validation on an external, withheld, dataset that is meant to assess classifier generalisability, the extended model achieved an F_1_-score of 91.14 ± 1.62%, outperforming its baseline counterpart which received a lower score of 88.30 ± 2.44%. We make further comparisons between the classifiers and discuss future improvements and directions, including utilising chemical and disease embeddings for visualisation and exploratory analysis of the DILI-positive literature.

## 1 Introduction

Drug-Induced Liver Injury (DILI) is a rare adverse drug reaction that can cause severe complications and, in some cases, may even prove fatal. The term is primarily used to signify the unexpected harm that a drug can cause to the liver. Virtually every class of medication can lead to hepatotoxicity, but the relative risk varies greatly between different drugs (David and Hamilton, 2010). For example, studies suggest that oral medications with doses higher than 50 mg/day and greater lipophilicity–thus those exhibiting higher hepatic metabolism–are more likely to cause DILI (David and Hamilton, 2010; Fontana, 2014).

Liver toxicity can be brought about in a predictable, dose-dependent manner when an individual is exposed to concentrations exceeding a drug’s toxicity threshold. This is known as intrinsic (or direct) DILI, has a relatively short latency period (hours to days), and is reproducible in animal models. The most often studied example of intrinsic DILI is acetaminophen (paracetamol), which accounts for about or more than half of acute liver failure (ALF) cases in the United Kingdom and United States (Katarey and Verma, 2016; Andrade et al., 2019). The majority of DILI cases, however, belong in the idiosyncratic (or indirect) variety which, as the name suggests, cannot be solely explained by the drug in question. This type of DILI is instead driven by a mixture of characteristics that are unique to the individual and their environment and tends to have a longer latency period following exposure (days to months) (Andrade et al., 2019). Idiosyncratic DILI is most prominently associated with antibiotics, and amoxicillin-clavulanate is the most commonly implicated drug in studies of European and American populations (Katarey and Verma, 2016).

Idiosyncratic DILI is, indeed, a rare occurrence, with two prospective population-based studies in France (Sgro et al., 2002) and Iceland (Björnsson et al., 2013) placing its crude annual incidence rate at 13.9 and 19.1 cases per 100,000 people, respectively. A retrospective study of the UK-based General Practice Research Database (GPRD) (de Abajo et al., 2004) reports a lower rate of 2.4 cases per 100,000 people, which is also in line with other studies from Sweden and the United States (Andrade et al., 2019). Out of those cases, an analysis of data coming from the Spanish DILI registry showed that about 4.2% progress to ALF (Robles-Diaz et al., 2014). This is in agreement with an incident rate of 1.61 cases per 1, 000, 000 people reported by another US-based study (Goldberg et al., 2015). Yet, despite its rarity, DILI remains one of the commonest reasons for the premature termination of drug development while also affecting already-circulating drugs, often leading to withdrawal from the market or issuing warnings and modifications of use (Katarey and Verma, 2016; Andrade et al., 2019). Therefore, the ability to reliably identify cases of DILI in the literature becomes critical, as such resources can aid both physicians in diagnosing the disease and researchers in, among other things, unravelling its mechanisms of action.

The identification of DILI-related literature is complicated by the heterogeneous and multifactorial nature of it. Typically, a drug causes hepatotoxicity directly, through its metabolites and/or due to possible subsequent inflammatory reaction. However, factors including pre-existing liver pathology, such as Hepatitis B or C, or non-alcoholic fatty liver disease (NAFLD), and chronic alcohol consumption can increase an individual’s susceptibility to DILI. Similarly, genetic factors are at play; different cytochrome p450 enzyme phenotypes can lead to either decreased metabolism of toxic drugs or accelerated production of toxic intermediates, and human leukocyte antigen (HLA) polymorphisms may cause enhanced immune-mediated mechanisms. Furthermore, the clinical presentation of the disease is broad, with symptoms that often mimic other acute and chronic liver diseases and, in the absence of diagnostic tests and biomarkers, diagnosis is primarily based on establishing a temporal association between drug exposure and symptom development which is assessed alongside clinical history, liver biochemistry, imaging, and in some cases biopsy (David and Hamilton, 2010; Katarey and Verma, 2016). This complex landscape makes DILI identification a challenging task, with the application of text-mining techniques on DILI-related literature (Cañada et al., 2017; Wu et al., 2021) remaining relatively sparse.

This work presents a contribution to the “Literature AI for DILI Challenge” which was part of the Critical Assessment of Massive Data Analysis (CAMDA) 2021 (http://camda2021.bioinf.jku.at). The aim of the challenge was to develop a classifier capable of identifying DILI-relevant papers. For that, we were given access to about 7,000 DILI-positive PubMed papers, referenced in the National Institutes of Health’s (NIH) LiverTox database (Hoofnagle et al., 2013), and a non-trivial reference dataset of around 7,000 DILI-negative papers. These originated from a larger collection of positive and negative corpora that was split in half to create a second dataset, similar in size with the one released (about 14,000 texts in total), that was withheld and used for final performance testing. We refer to this as “external validation” to distinguish it from the (internal) Nested Cross-Validation (NCV) that we perform. A second, smaller but more challenging, external validation dataset of 2,000 texts was also provided.

We built an analysis pipeline that combines Natural Language Processing (NLP) with small-molecule and disease similarities. We pre-processed and normalised the texts to exclude uninformative words and allow for comparisons to be drawn across them. Within each text, chemical and disease terms were annotated and extracted. We treated those as external features and applied a framework that is capable of capturing their similarity. For chemicals, we acquired vectors (embeddings) directly from the Chemical Checker (CC) (Duran-Frigola et al., 2020). For diseases, we first collected data that encode the relations that exist between them. These were sourced from the National Library of Medicine’s (NLM) Medical Subject Headings (MeSH) thesaurus (https://meshb.nlm.nih.gov/) and the Comparative Toxicogenomics Database (CTD) (Davis et al., 2021). We then followed a similar procedure as the one used in the CC to learn vector representations for diseases. Since, typically, a text is associated with multiple terms, an average chemical- and disease-vector (external feature vector) was calculated and attached to it. These, together with the normalised texts, were fed into a Neural Network (NN) classifier. To prevent over-fitting during training and to get an unbiased estimate of classification performance, we did hyperparameter tuning in a NCV scheme with 10 outer and 5 inner folds. During model evaluation, the extent to which external features alone are capable of distinguishing between the DILI-positive and negative texts was examined. Classifiers with and without the inclusion of external feature vectors were built and compared. During discussion, we explore drawbacks, point out future improvements, and focus on the potential impact of this work on facilitating DILI research.

## 2 Methods

This analysis is split in three consecutive stages, with each being dependant on the output of the previous ones. First, title and abstract pairs (texts) were collected and processed. This stage constitutes the NLP pipeline, which can be further split in two steps: text pre-processing, and chemical and disease term (concepts) annotation. At the second stage, drug and disease embeddings were learnt and an average drug- and disease-representation (external feature vector) was calculated for each text. Lastly, the NN classifiers were built and then trained, validated, and tested in a NCV scheme. The project has been developed in Python 3.9.10 and bundled as a package to provide ease of use and aid future development.

### 2.1 NLP Pipeline

#### 2.1.1 Text Pre-Processing

Titles and abstracts were first concatenated to form “full” texts. These were then provided as input to the Stanza NLP package (Qi et al., 2020), which was initialised with its tokenisation, lemmatisation, and Part-of-Speech (POS) processors. Stanza provides two biomedical Universal Dependencies (UD) models that are pre-trained on human-annotated treebanks. For this analysis, we used the option that is based on the GENIA corpus (Kim et al., 2003), as it is built on top of 2, 000 PubMed abstracts and was therefore thought to be a better fit for the (also PubMed-sourced) texts that we had at our disposal.

Each text was split to sentences and then words, and each word was mapped to its base form (lemma). We filtered out lemmas that were not nouns, verbs, adjectives, or adverbs. A list of stopwords was compiled by merging those included in the spaCy package in Python with the ones provided by PubMed (https://pubmed.ncbi.nlm.nih.gov/help/#help-stopwords). Subsequently, both stopwords and any lemmas that were less than 3 characters long were purged. As a result of those pre-processing steps, implicitly, the texts were also lowercase-normalised, and any numerals and punctuation marks were dropped.

#### 2.1.2 Concept Annotation

We queried PubTator Central’s (Wei et al., 2019) RESTful API to acquire annotations for chemicals and diseases. The tool performs concept disambiguation, which resolves conflicts when overlapping annotations are found and returns concepts normalised to their respective MeSH identifiers. We then counted the times each annotated term shows up within a text and calculated and assigned (text-specific) relative frequencies to them. In the code, the “PubTator” class is responsible for handling POST and GET requests to the server, processing the raw response data, and associating texts with annotated terms and their relative frequencies. At this step, raw, unchanged texts were used as input. As a result, the annotations we got back were incompatible and thus could not be utilised together with the pre-processed texts of the previous section.

To resolve this issue while also retaining clarity, the “UnivTextBlock” class was implemented in the code. The class provides a method for exporting processed text, in the sense that pre-processing has been applied and concept terms have been normalised, either by replacement with their MeSH identifiers or the broader concept category (that is, “disease” or “chemical”). To handle cases where a concept term spans across multiple words or is misaligned compared to the target word(s)– often the result of incorrect sentence segmentation or peculiarities in tokenisation–the code checks for degree of overlap. We observed good performance when demanding that the latter exceed a minimum threshold value of 90%.

### 2.2 External Feature Vector Generation

#### 2.2.1 Data Collection

We aimed to quantify disease and, separately, chemical similarity. First, we collected data from the MeSH thesaurus. Descriptors and supplementary concept records were downloaded in XML format. At the uppermost level, there are 16 categories which are further split in subcategories. Within each subcategory, descriptors are arranged in a hierarchical manner from most general to most specific. This results in a branching, tree-like structure. In the XML file, each descriptor is associated with one or multiple tree numbers, which represent paths taken from the root subcategory until the descriptor in question is reached.

These data were parsed into a dictionary that associates descriptors with their respective tree numbers. We selected for disease-descriptors by pruning those whose trees did not start with “C,” as category C is for diseases. Similarly, for chemicals, we filtered out descriptors that did not fall under category D, which contains drugs and chemicals. Supplementary concept records are not associated with tree numbers. Instead, they are mapped onto one or multiple descriptors. We parsed these relationships in a separate dictionary which we used to indirectly link supplementary concepts with the hierarchical structure described earlier.

We also collected data from the CTD, which associates diseases with chemicals, genes, pathways, and phenotypes. In this context, phenotypes refer to non-disease biological events and are expressed using the Gene Ontology (GO) as controlled vocabulary (Davis et al., 2021). As a result, disease-phenotype associations are further split into three datasets, one for each GO category: Biological Process (GO-BP), Cellular Component (GO-CC), and Molecular Function (GO-MF). In total, 6 datasets were downloaded from the CTD. Disease terms are expressed using MeSH identifiers and thus required no further processing.

#### 2.2.2 Concept Embedding Learning

After data collection, a procedure matching the one followed in the CC was used. This was applied separately to each dataset and consists of three consecutive steps: 1) turning the dataset into a corpus, 2) learning a (sparse) vector representation on it, and 3) embedding the latter into a lower-dimensional space. For the MeSH, concept terms are associated with tree numbers which represent paths. We traversed these paths starting from the root subcategory and saved each location on the tree as a word. A corpus was then built by repeating this process for all terms. For the CTD, concept terms are linked together through interactions with chemicals, genes, etc. We created a corpus by treating these interacting partners as words.

Corpora were mapped to a sparse vector space by applying a Term Frequency–Inverse Document Frequency (TF-IDF) transformation. Prior to that, frequent and infrequent words were dropped—that is, words associated with less than 5 or more than 80% of the terms. Following the transformation, any null (zero) vectors were purged alongside with their corresponding terms. An initial dimensionality reduction step was performed by means of truncated Singular Value Decomposition (SVD). Here, we kept the number of components that explained around 90% of the variance seen in the original data.

For learning the final embeddings, we ran the node2vec algorithm (Grover and Leskovec, 2016), with its default parameters, on a term similarity network. This produced 128-dimensional vectors. To create the similarity network, we first used cosine similarities to identify each term’s neighbourhood, which consisted of its 100 closest neighbours. We approximated the null distribution, empirically, by randomly sampling with replacement 100,000 pairs of terms and calculating their cosine similarities. This was used to map neighbour similarities to *p*-values. We built the network by merging the neighbourhoods together and assigning − log_10_ (*p*-values) as edge weights. We pruned any insignificant edges–that is, edges with weights less than or equal to 2– but demanded that each term be connected to at least three closest neighbours.

#### 2.2.3 Chemical Checker Embeddings

While chemical similarities from the CC need no further processing themselves, vectors are indexed by their InChIKeys. Since we normalise chemicals using MeSH identifiers, a mapping had to be created that would link the two. We queried ChemIDplus’s (https://chem.nlm.nih.gov/chemidplus/) API to retrieve MeSH terms and their respective InChIKeys and SMILES. We then used the MeSH thesaurus to associate MeSH identifiers with concepts and terms. However, a MeSH identifier usually points to multiple concepts–which typically consist of one or more synonymous terms–and sometimes more than one of those concepts or terms are associated with an InChIKey and/or SMILES. Therefore, to reliably translate from MeSH to InChIKeys, we also took into account the hierarchy of preferred concepts and terms that exists in the MeSH thesaurus.

In an attempt to further expand the number of MeSH terms that are associated with an InChIKey, we extracted relevant associations from the DrugBank database (Wishart et al., 2018) and merged them with those associations sourced from ChemIDplus. Furthermore, in order to enrich any sparse CC spaces, we utilised the CC signaturisers (Bertoni et al., 2021) to predict embeddings for chemical compounds that are not included in the original database. Here, the SMILES structural information–which had been acquired from ChemIDplus in the previous step–were given as input to the signaturisers.

#### 2.2.4 External Feature Vectors

##### 2.2.4.1 Generation

At this stage, disease embeddings have been generated–seven vector spaces in total; six from the CTD and another one from the MeSH–and chemical signatures have been retrieved from the Chemical Checker (25 spaces, augmented with an additional one generated from MeSH data). We first aimed to concatenate individual concept-specific spaces into one, so that diseases (and chemicals) were represented by a single vector space (and chemicals by a separate one). However, concatenation alone would not only lead to a considerable dimensionality difference between the resulting disease- and chemical-specific spaces (896 and 3,328 dimensions, respectively), but also potentially combine a great number of correlated features together, adding redundant dimensions to the produced space. Therefore, after concatenation followed a dimensionality reduction step using truncated SVD. We have carefully tuned this process to retain as much information as possible without introducing additional noise.

The vectors were not normalised or centred prior to concatenation and dimensionality reduction, as doing so in this particular case did not lead to significant differences. For diseases, we chose to concatenate the top two most dense CTD spaces (GO-BP and GO-MF; for rationale, see Results) and then performed truncated SVD to reduce them down to 103 dimensions, which explained about 90% of the original variance. Separately, we reduced the dimension of the MeSH space for diseases from 128 to 47 dimensions, which also retained about 90% of the variance of the original data. We concatenated the two reduced spaces to form the final 150-dimensional disease space. In a similar manner, for chemicals, we concatenated all the CC spaces and reduced them down to 265 dimensions. The MeSH space for chemicals was also reduced to 35 dimensions. In both cases, about 80% of the original variance was explained. By concatenation, the final chemical space was produced, which is 300-dimensional.

At this point, a single disease-specific (and a second chemical-specific) space exists. These are by no means related to the texts but instead encode similarities between the concepts that were extracted out of them earlier. In contrast, external feature vectors are meant to be text-specific and to capture the similarities between the texts, as these are encoded by the combinations of chemicals and diseases that show up in them. For each text, concept relative frequencies–already calculated during concept annotation–were used to calculate the weighted average for the disease embeddings and, separately, chemical embeddings. Concept embeddings that belong to terms mentioned infrequently within the text are, as a result, down-weighted compared to those associated with more frequent terms. The two (now text- and concept-specific) vectors were first normalised to unity and then concatenated to form the final external feature vector.

##### 2.2.4.2 Comparisons

For between-space comparisons, two measures were used: the Rank-Biased Overlap (RBO) (Webber et al., 2010) and Pearson correlation. The RBO is a top-weighted similarity measure that can be applied to non-conjoint ranked lists of indefinite length. The measure models the behaviour of a user comparing between two lists incrementally, at increasing depths, where at each depth a fixed probability of stopping exists.

To compare the similarity between two spaces, a procedure similar to the one described in the CC was followed (Duran-Frigola et al., 2020). First, the common concepts between the two spaces were identified. Then, for each concept, we computed a list of its 100 closest neighbours. We used cosine similarities and returned lists that were ordered by decreasing similarity. The similarity search was performed efficiently using the Faiss library in Python (Johnson et al., 2021). The two ranked lists were used to calculate a RBO similarity score (we set *p* = 0.7, making the search more top-weighted). The process was repeated for all common concepts, the similarity scores were aggregated, and their average value was calculated. This was used as the RBO similarity score for the space-pair.

To calculate Pearson correlations, we applied Canonical Correlation Analysis (CCA) on space-pairs to find orthogonal linear combinations (canonical variables) of their features that maximally correlate with each other. We kept the first three canonical variable pairs and, for each of them, calculated the Pearson correlation. By averaging those values together, the final space-pair correlation value was calculated.

### 2.3 NN Classifiers and Validation

**FIGURE 1:**
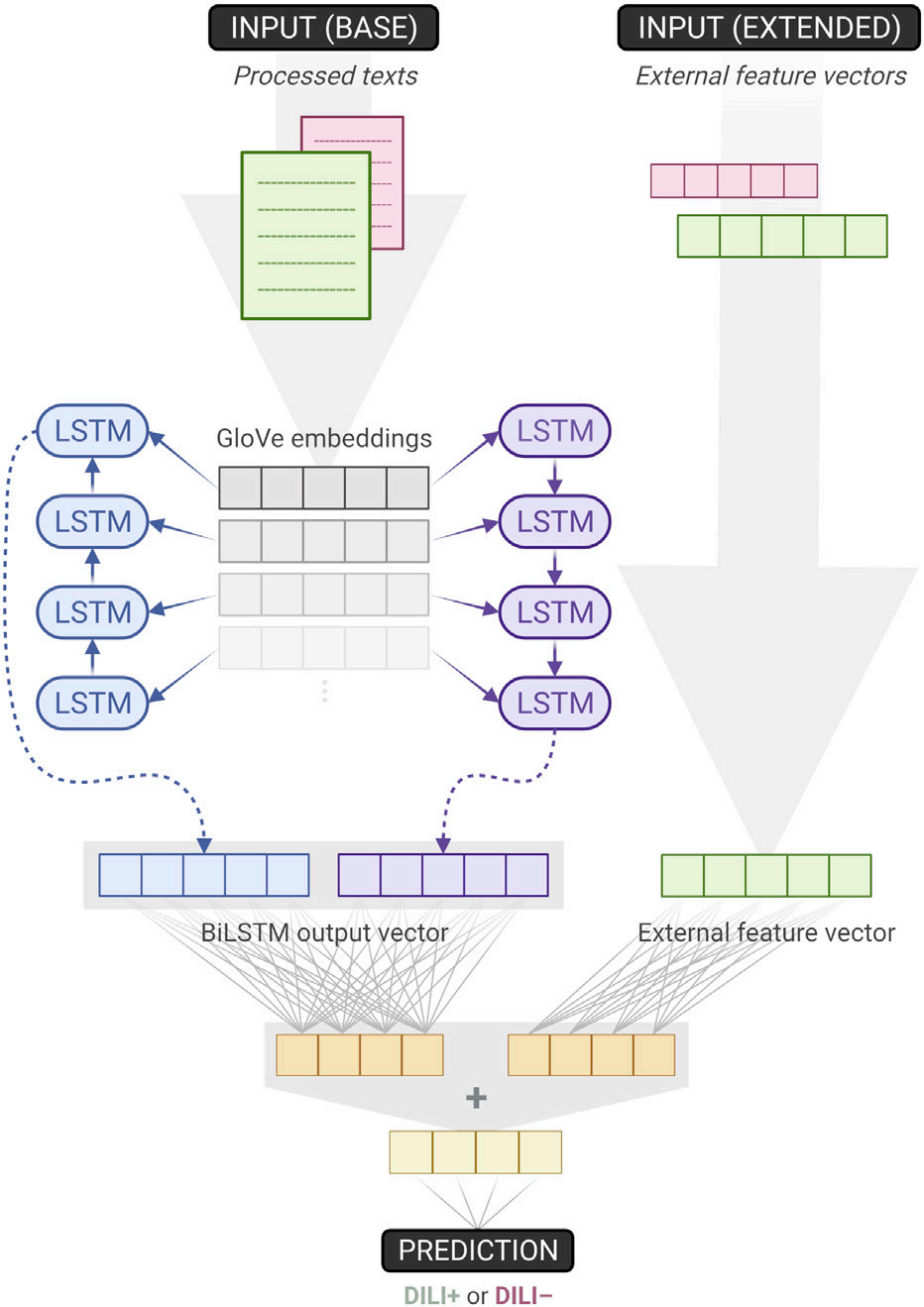
Overview of the baseline and extended classifiers. The former accepts texts as its single input, while the latter augments its baseline counterpart by also utilising external feature vectors. When training the baseline model, weights downstream the “base input”– on the left side of the figure–are learnt. Since the extended model is built on top of the baseline, those weights can be transferred and frozen, effectively remaining unchanged during training. As a result, for the extended model, only one dense layer’s weights have to be trained (Created with BioRender.com).

We developed two NN classifiers. The baseline classifier accepts processed texts as its single input. The extended classifier augments the baseline model by additionally taking into account each text’s external feature vector (Figure 1). First, the texts are fed into an embedding layer which has been initialised with GloVe vectors (Pennington et al., 2014). Then, these embeddings pass through a Bidirectional Long Short-Term Memory (BiLSTM) layer which is followed by a dense layer with ReLU activation. An output dense layer with sigmoid activation is used to compute the classification probability value.

For the extended classifier, the external feature vectors first pass through a separate ReLU dense layer that is chosen to have the same number of units as the one mentioned earlier. Thus, the outputs of those two dense layers can be added together before going through the same sigmoid output dense layer as the one used in the baseline model. This design choice is intentional, it introduces no additional hyper-parameters to optimise, and allows for both the baseline and extended models to be trained and tested within the same NCV scheme. We trained the 10 outer-fold baseline models (which were inner-fold winners), froze their weights, augmented and transformed them to extended models, and then repeated the training one more time. As a result, for the extended model, just a single dense layer’s weights needed to be trained.

During initial testing and tuning, it became apparent that using a Bi- instead of a Uni-LSTM layer and allowing for the text embeddings to be trainable consistently led to better performing models. Therefore, we did not optimise for those parameters. Nonetheless, hyper-parameter tuning was applied within a NCV scheme with 10 outer and 5 inner folds. We varied the embedding dimension ([50, 100, 200, 300]), Uni-LSTM units (32–96, with a step size of 16), dense layer units (192–320, with a step size of 32), and the learning rate ([10^–3^, 5 × 10^–3^, 7 × 10^–3^, 10^–2^]). During model training, we used a batch size of 32 and the Adam optimiser with binary cross-entropy as the loss function. For hyper-parameter tuning, we monitored validation loss. When training the extended model, a fixed learning rate of 10^–2^ was used.

Additionally, we observed that the models learn rapidly and usually start to overfit within the first 10 epochs, even with dropout and L1/L2 regularisation applied appropriately to the LSTM and dense layers. In fact, training for just one epoch tended to produce models performing similarly or better than those trained for longer. Thus, we chose to train for no more than one epoch. In this case, regularisation does not improve performance and was thus omitted (Komatsuzaki, 2019). We used Keras and the Bayesian optimisation algorithm in KerasTuner to build, train, and validate the NN classifiers and to perform hyper-parameter optimisation. To support NCV, the original KerasTuner code was subclassed and extended. We generate stratified k-folds through Scikit-learn’s (Pedregosa et al., 2011) “StratifiedKFold” function.

## 3 Results

### 3.1 Concept Embeddings

**FIGURE 2:**
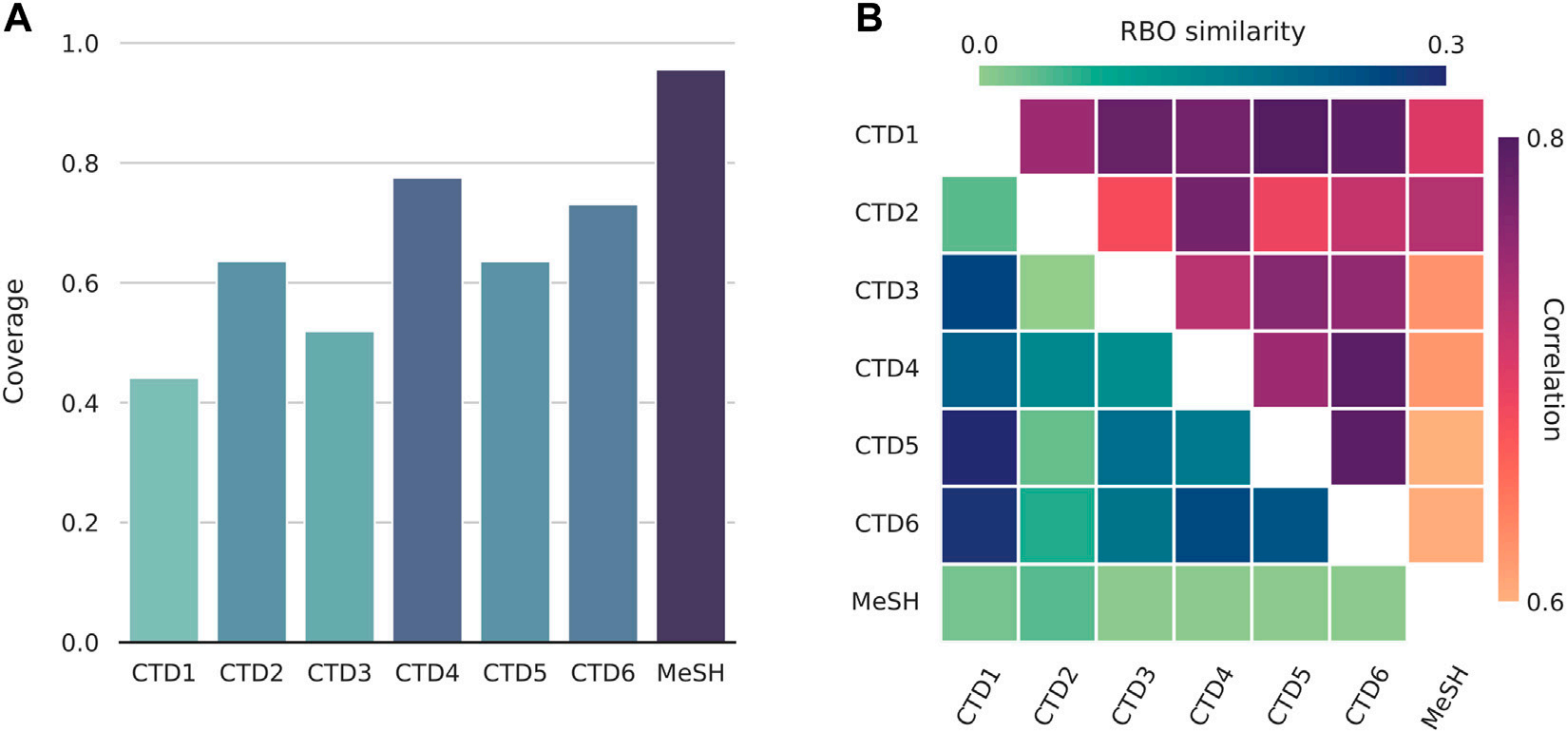
Comparisons between the different disease spaces. CTD1-6: Genes, Chemicals, Pathways, GO-BP (Biological Process), GO-CC (Cellular Component), and GO-MF (Molecular Function). **(A)** Disease term coverage for the 6 CTD (Comparative Toxicogenomics Database) spaces and MeSH (Medical Subject Headings). The latter, together with GO-BP and GO-MF, are the most enriched spaces, encoding about 96%, 78%, and 73%, respectively, of all the disease terms found in text. **(B)** RBO (Rank-Biased Overlap) similarities and Pearson correlations between disease spaces. Although the two are not directly comparable, they seem to be in good agreement with each other, with MeSH being the least and CTD1 (Genes) the highest correlated space, in general.

We first examined the degree of term coverage for the different concept spaces (Figure 2A). Chemical and disease terms found in the texts have already been extracted and collected. However, there are terms missing in some of the spaces. This is either due to the term not being present in the data that was used to construct the spaces in the first place or a result of the TF-IDF word filtering steps that were applied afterwards. Missing terms are represented as null vectors. For diseases, MeSH is the most enriched space (96% coverage), followed by the CTD’s GO-BP and GO-MF (coverage of 78% and 73%, respectively). Since we normalise terms to the MeSH vocabulary, the MeSH space is expected to be the most enriched.

For chemicals, the CC spaces appear to be equally enriched, with a coverage of about 60%. The MeSH space for diseases has a higher coverage of 84%. The uniformity that is observed across the different CC spaces can be attributed to, and also supports, the usage of the CC signaturisers. These fill in the gaps of missing molecular signatures; typically, CC spaces tend to differ considerably in terms of their sizes (Duran-Frigola et al., 2020). Notably, a concept space with lower term coverage does not necessarily translate to external feature vectors with reduced text coverage. The latter are (text-specific) linear combinations of concept vectors and the coverage of that space is, thus, also affected by the combination of terms that show up in each particular text as well as their relative frequencies.

We then calculated the RBO similarity measures and Pearson correlations across the different pairs of disease spaces (Figure 2B). The two measures are in good agreement with each other. As expected, given that the rest of the spaces are based on the CTD-sourced datasets, the MeSH space tends to be the most dissimilar one, followed by the CTD’s Chemicals space. On the other end, CTD Genes is highly correlated with most other CTD spaces. We created similar plots to compare between the chemical spaces and, for the CC spaces, observed a similarity and correlation profile that matched the one provided and discussed in the original publication (Duran-Frigola et al., 2020).

We utilised both coverages and correlations when selecting for the disease spaces and, separately, chemical spaces to concatenate. For diseases, we chose the top three enriched spaces (MeSH, and CTD GO-BP and GO-MF). When seen as a group, these are strongly correlated with the rest of the CTD spaces. We chose to concatenate all chemical spaces together. The premise here is that concatenation between spaces with largely different term coverages might introduce unwanted noise later, during the dimensionality reduction step (see *Methods*). This is of concern for diseases, where coverage ranges from as low as 44% to a highest score of 96%, but not for chemicals, where it remains virtually unchanged across the CC spaces.

### 3.2 External Feature Vectors

**FIGURE 3:**
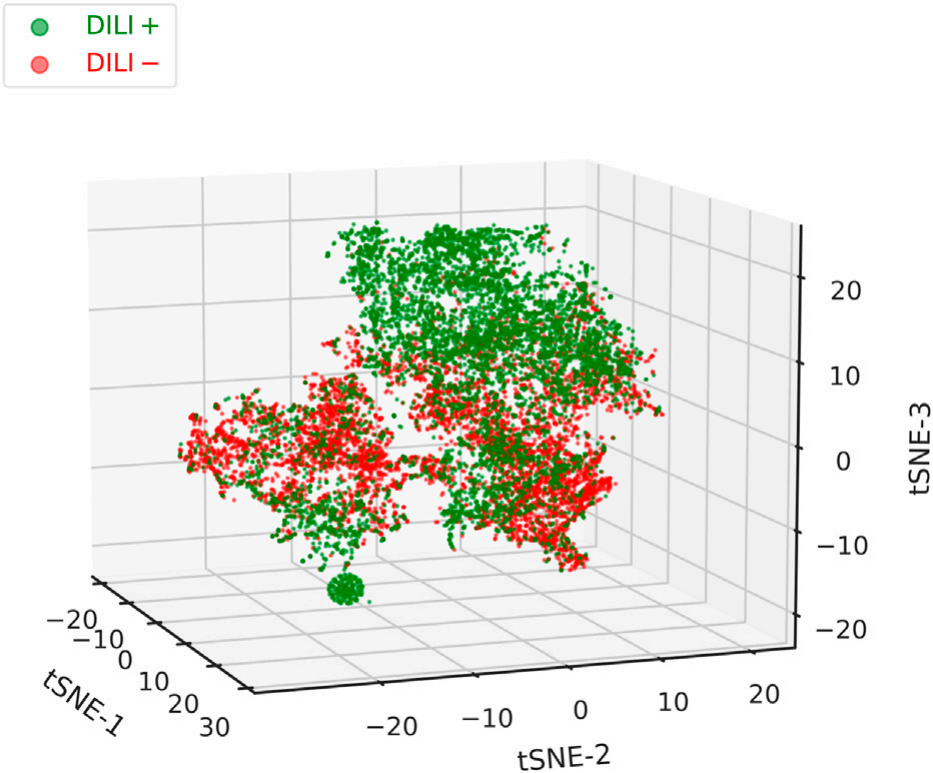
3D t-SNE plot of all the non-zero external feature vectors (which combine both chemical and disease embeddings). A good degree of separation can be observed between the DILI positive and negative texts, with the former clustering cleanly on the top-right corner of the plot. A second dense cluster is formed at the bottom-left corner. Between them, in the middle part of the plot, reside both positive and negative texts that did not form distinct clusters. While feature vectors can improve classification performance, they tend to be sparse and should, therefore, not be used as the single input to the classifier.

We were also interested in assessing the extent to which the external feature vectors are capable of capturing the differences between DILI positive and negative texts. Ability to do so, at this stage, would provide strong evidence of their suitability to be used as additional inputs to the (extended) classifier. First, we normalised the vectors, performed Principal Component Analysis (PCA), and kept the first 15 components. We then applied t-Distributed Stochastic Neighbour Embedding (t-SNE)– a technique for visualising high-dimensional datasets in two or three dimensions–to produce a 3D t-SNE plot (Figure 3). The method is particularly suited for our purposes here, as it preserves the local structure of the data (van der Maaten and Hinton, 2008). Throughout this work, as is usual in NLP, we are working with cosine similarities. However, cosine distances are not invariant to mean-centring–which PCA implicitly performs–and will be affected and distorted. In contrast, euclidean distances are mean-centring invariant. By normalising the data first, we enforce a monotonic relationship between cosine and euclidean distances, which we later exploit by using euclidean distances in the t-SNE plot (Korenius et al., 2007).

In the plot (Figure 3), a good degree of separation can be observed between the DILI positive and negative samples. Positive texts tend to cluster in the upper-right and also form a tight cluster in the lower-left corner. Between those, both positive and negative texts reside, forming largely overlapping clusters. It should be pointed out that concept vectors were learnt completely separately from, and are in no way connected with, the classification of texts in the two classes. As such, the acceptable clustering performance seen here should be attributed in: 1) similar chemicals and/or diseases appearing within each class, 2) dissimilar chemicals and/or diseases appearing between classes, 3) unique chemical, disease, and chemical-disease combinations dominating in each class. This might be worth further investigation but, for the moment, makes an appealing case for the usefulness of the external feature vectors as a means for improving classification performance.

### 3.3 Classification Performance

We compared between the baseline classifier, which uses texts as its sole input, and the extended one that also accepts external feature vectors. As we are interested in the balance between precision and recall, we used the F_1_-score as performance measure. During internal validation, macro F_1_-score (average of per-class scores) was calculated. For external validation, micro scores (calculated over the entirety of the predictions, irrespective of classes) are reported. During initial tuning and testing, we observed that the baseline model performs optimally with the usual classification threshold of 0.5 but, for the extended model, a higher threshold of about 0.7 leads to unchanged or improved performance, depending on the validation dataset used. We set these, seemingly arbitrary, thresholds at the beginning of the NCV procedure and evaluated their suitability afterwards. Alternatively, a more elegant approach would treat the classification threshold as a hyper-parameter to be optimised in the inner NCV folds.

**FIGURE 4:**
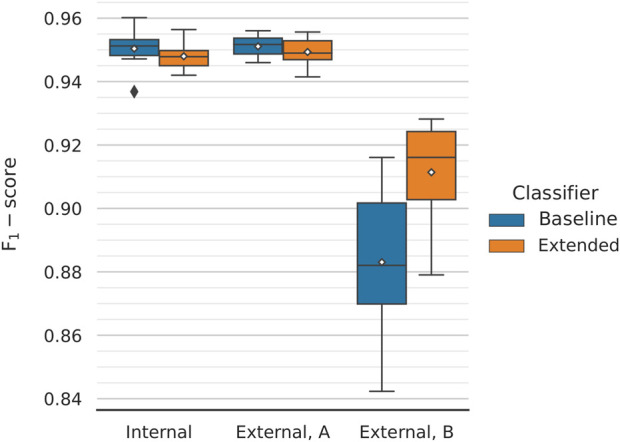
Classification performance comparison between the two models for different validation datasets. The performance across 10 folds is being reported. Mean values are annotated with white diamonds. Both classifiers perform virtually identically during internal validation (macro F_1_-scores of 95.04 ± 0.61% and 94.80 ± 0.41%, respectively) and equally well when tested on the first external dataset (micro F_1_-scores of 95.11 ± 0.34% and 94.93 ± 0.48%, respectively). The models are not overfitting. There is a significant drop in performance when testing on the second external dataset (meant to assess the classifier’s generalisability). However, the extended model performs considerably better, with a (micro) F_1_-score of 91.14 ± 1.62% compared to the baseline’s 88.30 ± 2.44%.

We plotted the average performance across the 10 outer folds (Figure 4). During internal validation, the baseline and extended models performed virtually identically, with F_1_-scores of 95.04 ± 0.61% and 94.80 ± 0.41%, respectively. Evaluating the models on the first external dataset, which closely resembles those used for training and internal evaluation, painted a similar picture; most importantly, this provides proof that the training procedure we utilise does not lead to overfitting. In this case, baseline and extended models achieved scores of 95.11 ± 0.34% and 94.93 ± 0.48%, respectively. We observed a drop in performance, which affected both models, when testing on the second external dataset. However, the extended model managed to outperform the baseline model by a considerable margin; the former achieved an F_1_-score of 91.14 ± 1.62% compared to the baseline’s 88.30 ± 2.44%. The extended classifier also seems to produce lower dispersed scores, which becomes especially pronounced during the second phase of external validation. For this, the classifiers are tested against texts that are not title-abstract pairs; for the positive set, LiverTox (Hoofnagle et al., 2013) annotations are included instead. The better performing extended model can then be assumed to generalise better on texts that follow a different feature distribution than the one the classifier was trained on.

**FIGURE 5:**
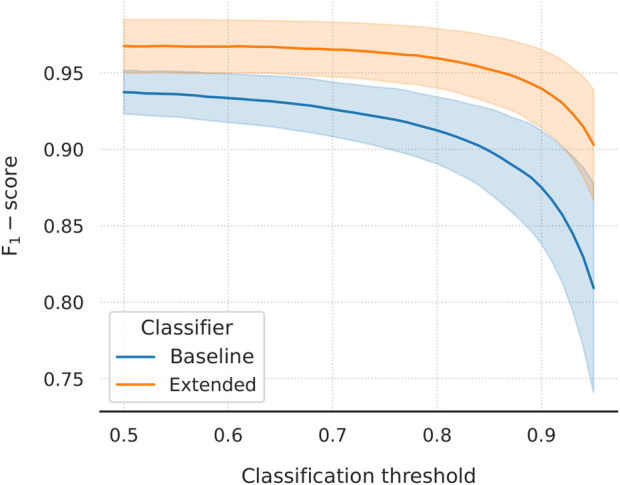
Macro F_1_-score as a function of classification threshold varied in the domain [0.5, 0.95]. Mean values and standard deviations are plotted. The extended model outperforms the baseline at every threshold value. There is a clear difference in slope, with the baseline curve being steeper throughout the domain. Learning from the external feature vectors has pushed the extended classifier to be more confident in its predictions. In turn, using the same threshold for both models will lead to (at least) one of them under-performing. A higher threshold is desirable, as it tends to improve the performance of the models on the more challenging second external dataset.

Lastly, we evaluated the choice of threshold for the two classifiers. Within each outer fold, we varied the threshold between 0.5 and 0.95 and calculated the (macro) F_1_-scores (Figure 5). When compared at the same classification threshold, the extended model consistently outperforms its baseline counterpart by a small margin at thresholds closer to 0.5, which incrementally grows larger at higher thresholds. This implies a difference between the slopes of the two curves which is, indeed, there to be seen: the baseline curve is steeper at each threshold value compared to the extended one. The inclusion of the external feature vectors has resulted in the extended classifier being more confident in its predictions, which is reflected in the probability scores being pushed closer to the limit points of the [0, 1] interval (and a lower binary cross-entropy validation loss, too). It is desirable to set the threshold to a higher value, as doing so can improve–sometimes considerably–the classification performance on the second external dataset. Higher thresholds, however, might hurt the performance on the internal and the first external validation datasets. For the extended model, choosing a threshold in the range of 0.5–0.7 leads to virtually unchanged F_1_-scores, a behaviour not followed by the baseline model. With this in mind, the choice of thresholds for the two classifiers seems to be near-optimal.

## 4 Discussion

In this work, we set out to develop a classifier that is capable of accurately distinguishing between DILI-positive and negative literature. To do this, we began by utilising word embeddings as a means of capturing the semantic similarities between individual words and, subsequently, the texts these appear in. However, word embeddings learnt directly on DILI-related (or other clinical or biomedical) literature should not be expected to capture the similarities between diseases and chemicals. Ultimately, while a variety of different approaches exist, methods for learning (word) embeddings rely on context—that is, the entities (in this case words) that tend to appear around a target one. As an example, GloVe embeddings encode linguistic and/or semantic similarities of words by taking into account co-occurrences (Pennington et al., 2014), and the word2vec algorithm either predicts a target word given those around it (Continuous Bag-of-Words Model; CBOW) or tries to predict surrounding words based on the target (continuous Skip-gram model) (Mikolov et al., 2013). Then, it becomes apparent that no appropriate context exists in free text for meaningful relations to be encoded for either chemicals or diseases. For the former, such context could instead be shared indications, protein targets, or adverse effects; for the latter, common disease-associated genes–including how these might relate through GO terms–or shared therapeutic chemicals. This is the rationale behind the usage of concept embeddings here.

Concept embeddings, turned into text-specific external feature vectors, however, present a challenge when utilised alone for classification. For the DILI-positive class, 93% and 86% of the texts have been annotated with at least one disease and chemical term, respectively. In the negative class, these percentages shrink down to 78% for diseases and 56% for chemicals. The lack of annotated concept terms can be attributed to: 1) failure to annotate terms that exist in the text (false negatives), or 2) genuine lack of terms (true negatives), or 3) lack of terms in the title and/or abstract but presence in the full text (true negatives in the context of the challenge but false negatives in the broader sense). Because of the first and last points, filtering out texts with no annotated concepts as DILI-negative would be problematic. Instead, combining concept with word embeddings enables the classifier to make informed decisions even when no chemicals or disease terms have been identified. As a result, acquiring full texts when no concept terms are included in the title and abstract could have the potential to further improve classification performance.

Interestingly, the inclusion of chemical and disease embeddings leading to improved F_1_-scores was not a behaviour shared between the two external validation datasets (Figure 4). While the extended classifier outperformed the baseline during the second round of external validation, the two models behaved equally well on the first. Moreover, both suffered reduced performance when moving from the first to the second round. The latter can be attributed to the different nature of the data. Although the first external validation dataset is a collection of paper title and abstract pairs–similar to those used for model training, and internal evaluation and testing–the second comprises of a combination of LiverTox (Hoofnagle et al., 2013) DILI-positive annotations and a set of non-trivial DILI-negative texts. Therefore, it is meant to assess the robustness of the classifiers on unseen texts that should also be expected to follow a different underlying feature distribution. That explains the drop in performance observed during the second round of external validation and, partially, the difference (or lack thereof) in behaviour between the two models. The extended classifier seems to generalise better on unseen data, a trend that is captured by the second but not the first external dataset.

**FIGURE 6:**
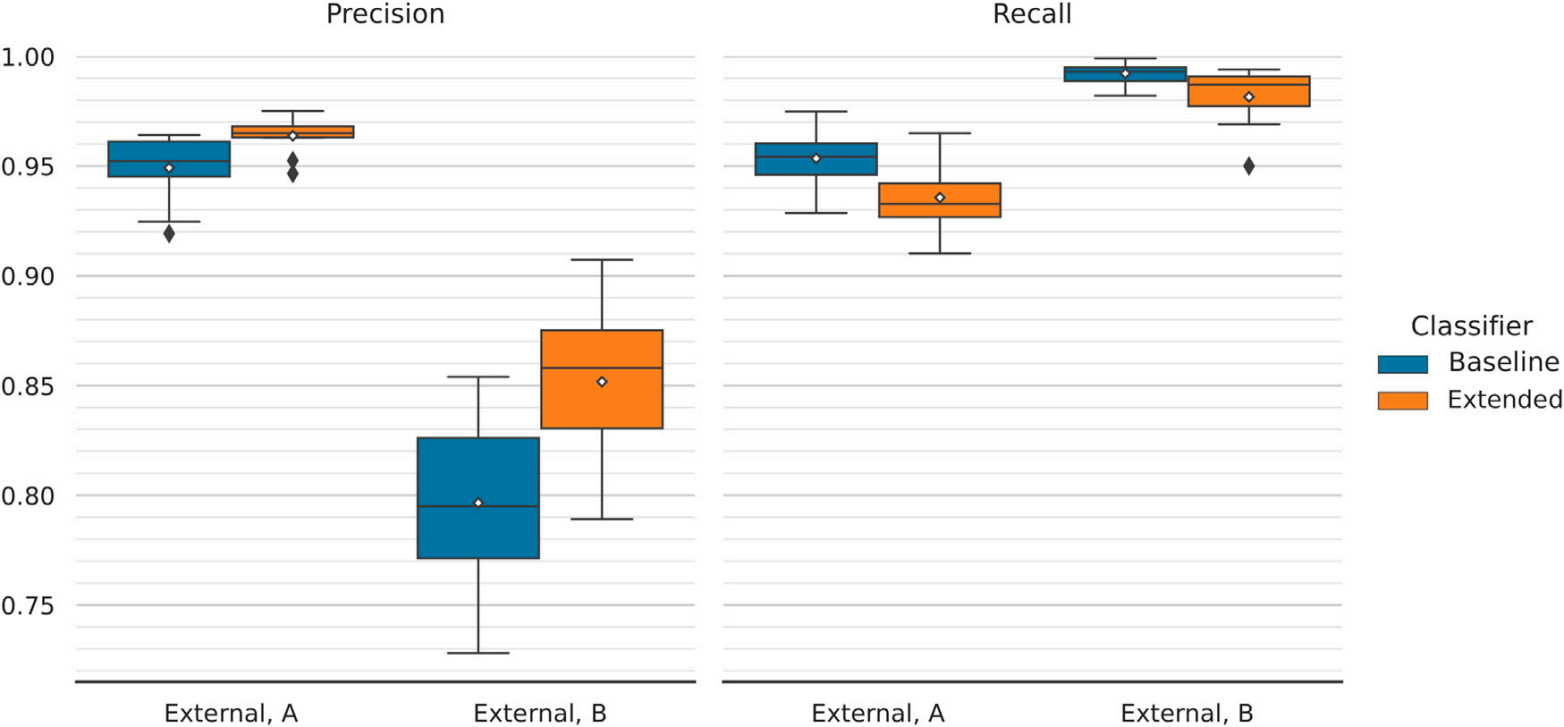
Comparison of precision and recall scores between the two models during external validation. Classification performance is reported across 10 folds and mean values are annotated with white diamonds. Overall, moving from the first to the second external dataset results in significantly decreased precision and moderately improved recall (with the latter approaching unity). No notable performance difference is observed between the models, with the exception of precision achieved on the second validation dataset (assessing generalisability). There, the extended model gets a higher score of 85.17 ± 3.63% compared to 79.66 ± 4.20% of its baseline counterpart. On the same data, extended and baseline models achieve recall scores of 98.15 ± 1.37% and 99.22 ± 0.51%, respectively.

Further to that, the improved behaviour of the extended model during the second round of external validation is a result of the threshold moving that was performed during model performance tuning. We have showed that the extended classifier, in contrast to the baseline model, maintains a high, almost unchanged, F_1_-score over a large range of increasing classification thresholds (Figure 5). This captures the expected behaviour of the model on data of similar nature to the first external dataset. We hypothesise that this dataset includes a more trivial negative set of texts compared to the second. Because of that, making predictions on the latter leads to a rise in false-positives, which is reflected in the steep drop in precision that is observed (while recall improves moderately; Figure 6). In this case, the extended classifier performs better because of its ability to make more confident predictions (probabilities pushed towards unity), which justified and allowed for the selection of a higher classification threshold in the first place. That further supports the observation that the inclusion of drug and disease embeddings has resulted in a more robust classifier. Training with a more challenging negative set of texts might further improve classification performance. Additionally, selecting an even higher threshold could trade increased precision for reduced recall–a viable option given that recall already approaches unity.

The additional information that we either generate or collect about disease, chemical, and text similarity can also prove valuable for the purposes of visualisation and exploratory analysis. Similar to the t-SNE plot that we provide in this study (Figure 3), texts could be further clustered together based on the combination of chemicals or, alternatively, diseases that occur therein–a process that inherently takes into account concept similarities, too. Alternatively, average chemical similarities could be calculated against drugs that are already known to cause DILI, for example with the help of the DILIrank dataset (Chen et al., 2016). These could be used to rank DILI-positive texts from most (high similarity to known DILI-related drugs) to less promising, as well as annotate them separately on the t-SNE plot so that their neighbourhoods can be identified and further explored. This is one of the most exciting future prospects of this work.

**FIGURE 7:**
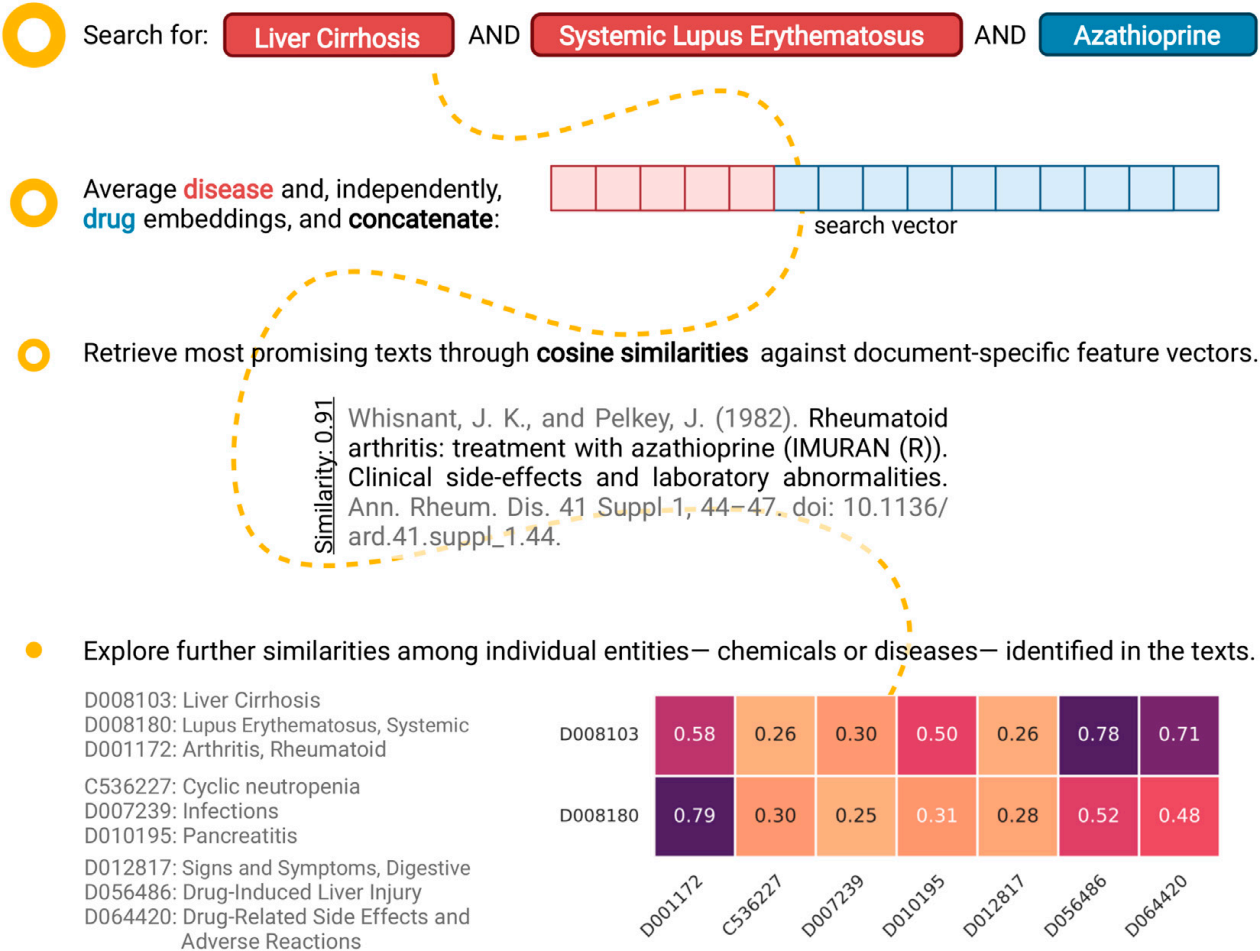
An example case of exploratory analysis: retrieving non-trivially similar papers. Identifying documents that co-mention “Liver Cirrhosis (LC),” “Systemic Lupus Erythematosus (SLE),” and “Azathioprine” presents an interesting challenge, as that specific combination does not exist in the positive training set. Notably, searching through the complete (positive and negative) training set using our proposed approach uncovers a non-trivially similar text as the best hit (cosine similarity of about 0.91). Further looking into the similarities between the query and in-text diseases (along the *x*-axis; sorted by decreasing in-text frequency) reveals that, while no overlapping terms exist, a link between SLE and rheumatoid arthritis has been identified (similarity of 0.79). Likewise, the close connection between LC, and Drug-Induced Liver Injury (DILI) and adverse drug reactions has been captured (similarities of 0.78 and 0.71, respectively) (Created with BioRender.com).

In support of the latter, we utilised the text-specific external feature vectors that have already been calculated to retrieve non-trivially similar texts. Searching against an existing text that is included in the positive training set typically leads to the identification of a large number of highly-similar texts with overlapping terms (diseases and/or chemicals). While this is desirable behaviour, the existence of multiple overlapping terms does not make for an appealing case of “non-trivial similarity.” For that reason, we created a dummy query feature vector which represents a text that mentions Liver Cirrhosis (LC), Systemic Lupus Erythematosus (SLE), and azathioprine. This is a combination that does not exist in the positive set of the training data. Ranking, in terms of cosine similarity, both DILI-positive and negative training texts against the aforementioned query vector reveals that the best hits mention azathioprine but none of the disease terms. When looking further into the top-scoring text (score of about 0.91; Figure 7), we observe a high degree of similarity between the diseases that are mentioned in-text and the ones that were queried. Notably, SLE is linked with rheumatoid arthritis with a similarity score of 0.79, and LC with DILI and adverse drug reactions with scores of 0.78 and 0.71, respectively. In a similar manner, non-obvious associations can be brought forward for further consideration; such connections are also captured on the t-SNE plot (Figure 3).

Taken together, these points make a strong case for the inclusion of chemical and disease embeddings in the proposed algorithm. First, they allow for the classifier to make more decisive predictions which, in turn, places the optimal (as in, the one that improves on or, at worst, leaves unchanged the performance of the classifier on all datasets) classification threshold above 0.5. As a result, the extended classifier generalises better on alternatively-sourced texts compared to the baseline model (Figure 4). We would expect this behaviour to translate similarly to imbalanced datasets, though this case was not explicitly tested here. Most notably, though, the proposed pipeline can facilitate the visualisation and further exploratory analysis of the identified literature. Ultimately, an automated classification procedure can only do so much before human intervention and reasoning is required. In this regard, the potential to further cluster publications based on the similarities of the in-text mentioned drugs and/or diseases can both help uncover hidden relations that exist between them and speed up the overall process of accumulating enough “incriminating” evidence implicating a particular drug with DILI (and the conditions under which this happens).

## Data Availability

The code and datasets (directly uploaded or with links to the original sources) used in this study can be found on the GitHub repository: https://github.com/pokedthefrog/camda2021-dialogi.
